# A national survey of practice for the emergency fixation of testis

**DOI:** 10.1308/rcsann.2023.0101

**Published:** 2024-02-16

**Authors:** Paediatric Surgery Trainee Research Network (PSTRN) and British Urology Researchers in Surgical Training (BURST)

**Affiliations:** Norfolk and Norwich University Hospitals NHS Foundation Trust, UK

**Keywords:** Testicular torsion, Orchidopexy, Genital diseases, Male, Testicular fixation, Scrotal exploration, Acute scrotum

## Abstract

**Introduction:**

Scrotal exploration for suspected testicular torsion is a common emergency procedure in the United Kingdom (UK). There is no universally agreed practice for how the testis should be fixed, or whether a nontorted testis should receive fixation. This survey aims to describe the methods used for emergency scrotal exploration and testicular fixation in the UK.

**Methods:**

An online survey was distributed to urologists, general surgeons and specialist paediatric surgeons in approved NHS trusts, and via the email lists of collaborating organisations. The survey questioned surgeons on their operative management of a variety of common diagnoses encountered during scrotal exploration using multiple choice and free-text answers.

**Results:**

A total of 340 responses were received from 83 institutions. Respondents included urologists (consultants, 33%; trainees, 24%), paediatric surgeons (consultants, 12%; trainees, 16%) and general surgeons. In cases of torsion, respondents predominantly perform sutured fixation (74%); however, sutureless dartos pouch fixation was used frequently (37%) by paediatric surgeons. The finding of ‘bell-clapper’ anatomy without torsion prompts 69% of respondents to undertake sutured fixation, but alternative nontorsion diagnoses frequently prompt use of sutureless methods (53–66%).

**Conclusion:**

This study is the largest survey of methods for emergency scrotal exploration and describes current UK practice. The majority of surgeons prefer sutured fixation in cases of torsion and/or bell-clapper anomalies, and sutureless methods in the absence of it.

## Introduction

Scrotal exploration for suspected testicular torsion is one of the most common emergency procedures performed in the United Kingdom (UK). When the diagnosis of testicular torsion is suspected, emergency surgery is undertaken in an attempt to salvage the testis from irreversible ischaemia. The procedure is performed by urologists, general surgeons and, in children presenting to tertiary centres, by specialist paediatric surgeons. As a routine emergency procedure that is frequently performed out of hours, it is often trainee-led.^1–3^

Alternative diagnoses, other than testicular torsion, may be encountered on the operating table, including epididymitis, torted testicular appendage (TTA) and varicocoele. Testicular fixation can be performed with sutures, by fixing at one or more points with absorbable or nonabsorbable suture materials, or by sutureless methods such as placement in a sub-dartos pouch, or eversion of the tunica vaginalis (TV), to encourage the formation of adhesions to fix the testis.^4^

There is no universally agreed practice on how to fix a testis to prevent further torsion and no universal agreement on whether a testis that is not found to be torted intraoperatively should receive fixation.

Other aspects of the procedure can also vary greatly; for example, the incision, method of closure and decision about whether to explore the contralateral side. The recent British Urology Researchers in Surgical Training (BURST)/British Association of Urological Surgeons (BAUS) FIX-IT statement outlines an expert consensus on best practice for a typical patient undergoing emergency scrotal exploration, as well as highlighting key differences in the surgical approach to adults and children with testicular torsion.^5^

This study aims to describe the methods used for emergency scrotal exploration and testicular fixation across the UK, and assess the variations between specialties, seniority of operating surgeons and differences in approach to adults and children.

## Methods

An online survey of practice was developed jointly by the Paediatric Surgical Trainee Research Network (PSTRN) and BURST collaboratives using Forms (Microsoft, 2018). The survey consisted of 7 sections with a total of 77 nonrandomised questions. Branching logic was applied to reduce the complexity of the question items; therefore, participants were unlikely to answer all 77 questions. In total, 21/77 main stem questions were mandatory, with progress prohibited until all were answered, meaning all questionnaires were eligible for analysis. Participants were allowed to review and change their answers prior to survey submission (Supplementary Appendix available online).

Health Research Authority approval was gained for distribution in England, Scotland, Wales and Northern Ireland. Eligible respondents were consultant and training grade surgeons for whom scrotal exploration forms part of their clinical practice. The survey was open from January to November 2021 and was primarily distributed by the local research and development (R&D) departments of approved NHS trust sites with the assistance of collaborators within individual centres. Distribution was increased via the email lists of collaborating organisations: PSTRN, BURST, British Association of Paediatric Surgeons (BAPS), British Association of Paediatric Urologists and Trainees in Paediatric Surgery. Questionnaires from non-UK respondents on these email lists were permitted. Participants contacted through R&D departments and those on the PSTRN and BURST mailing lists received a single reminder. The survey was advertised on social media and at society meetings but the link to the survey was not available through these mediums to avoid completion of the survey by ineligible individuals.

Responses were voluntary with no incentives, and informed consent was gained by distributing participant information sheets and General Data Protection Act statements with the survey link. No identifiable data were collected, and electronic survey results were stored securely. To prevent duplicate responses, recipients were asked in the invitation email to answer only once, and in the survey to provide three specified digits of their General Medical Council (GMC) registration number, to prompt review if similar answers are provided in more than one response with the same three digits. Results are reported using the Checklist for Reporting Results of Internet E-Surveys (CHERRIES) framework.^6^

The main outcome of interest was the variation in testicular fixation method depending on the pathology and the operating specialty.

Respondents were grouped according to their speciality: urologists, specialist paediatric surgeons and paediatric urologists (referred to as paediatric surgeons henceforth for clarity), and general surgeons. Subgroup analyses of reported seniority (consultant, trainee/fellow) were also performed. Data are absolute numbers and percentages with 95% confidence intervals (CI) or mean with 95% CI as appropriate.

## Results

The survey was distributed to a total of 31 UK NHS trusts via R&D departments, and the mailing lists of the above organisations. The number of recipients was estimated by: (a) enquiring with the NHS trust R&D departments; and (b) counting the number of people on email distribution lists, resulting in an estimated 3,100 recipients (546 from NHS trusts, 2,222 from the BURST mailing list, and ∼330 from other mailing lists). In total, 340 responses (11%) were received and included from surgeons working in 83 different institutions. One response was identified as a possible duplicate, owing to identical GMC digits and centre. This response is included in the data presented. A sensitivity analysis was performed excluding this questionnaire, which demonstrated no significant change to the results.

### Demographics and caseload

The highest proportion of respondents were urologists (193/340, 57%), followed by paediatric surgeons (117/340, 34%) and general surgeons (30/340, 9%) (Supplementary Figure S1 available online).

The majority of respondents (270/340, 79%) report having performed more than 26 scrotal explorations in their career, and in the past year 262/340 (77%) had performed between 0 and 10 as the lead operator (Supplementary Table S1 available online).

Most surgeons indicate that they undertake surgery for paediatric patients with acute scrotal pain, although 52/340 (15%) state that they have an exclusively adult (≥16 years) practice. Some 113/340 (33%) report a lower operative age limit of 1 month and 93/340 (27%) have an upper limit of 16 years. When categorised by specialty, the majority of urologists and general surgeons have a lower age limit of between 1 and 5 years, with no upper limit, whereas (91%, 107/117) paediatric surgeons operate with no lower age limit and the majority (81/117, 69%) have an upper limit of 16 years.

### Surgical approach

Of 324/340 respondents using a single technique for all situations, the midline incision (177/324, 55%) followed by transverse hemiscrotal incision (132/324, 41%) were favoured. When divided by specialty (excluding the 16 that varied their approach by age/puberty) urologists prefer a midline approach (150/181, 83%), paediatric surgeons a transverse approach (102/117, 87%) and general surgeons either approach (16/29 midline vs 12/29 transverse, 55% vs 41%) (Supplementary Figure S2 available online). Overall, the paramedian craniocaudal incision was used infrequently (11/340, 3%). Seven respondents (2%) report that they vary their approach based on pubertal stage and nine (3%) vary their approach by age.

### Fixation of a nontorted testis

**Figure 1:**
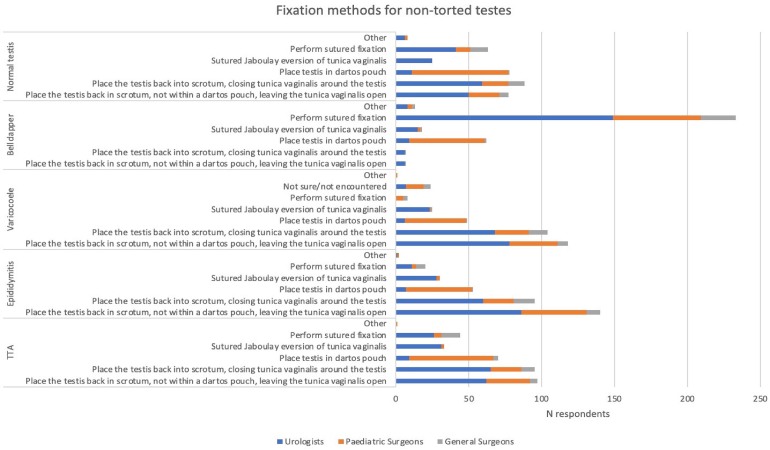
Management preference on finding a nontorted testis

During exploration, if a pathology other than testicular torsion is encountered (such as TTA, epididymitis, varicocoele or normal testis), the majority of respondents stated they would not undertake suture fixation of the symptomatic testis. On expression of the total responses for all nontorted pathologies as a range, 88/340 to 104/340 (26% to 31%) would opt to close the TV and restore normal anatomy, compared with those who favour techniques that may induce sutureless fixation (181/340 to 224/340, 53% to 66%) such as leaving the TV open, dartos pouch or Jaboulay eversion of the TV. If a bell-clapper deformity^7^ is encountered, 233/340 (69%) opt for a sutured fixation (Figure 1).

A three-point fixation with nonabsorbable monofilament suture is the most common preference when a sutured fixation answer was chosen for any diagnosis. Three respondents describe using a two-point fixation for nontorted testes with a bell-clapper deformity.

The use of a fixation method between the consultant and trainee groups were similar, apart from in the scenario in which there was a normal testis, where trainees were more inclined to use a dartos pouch (29% [95% CI 26–31] vs 18% [95% CI 16–21]) and less likely to place back in the scrotum, leaving the TV open (17% [95% CI 14–20] vs 27% [95% CI 25–30]), compared with consultants.

Paediatric surgeons were more likely to use dartos pouches in all scenarios compared with their adult specialist counterparts: paediatric, 45% (95% CI 39–50); urologists, 4% (95% CI 3–6); and general surgeons, 5% (95% CI 3–7).

On finding a nontorted testis, 202/340 (59%) will not perform a contralateral procedure under any circumstances. Contralateral fixation is undertaken by 125 (37%) of respondents for a bell-clapper deformity, by 35 (10%) for a normal ipsilateral testis with no evident pathology and by 11 (3%) respondents for other pathologies in addition such as TTA, varicocoele and epididymitis.

### Management of a torted testis

On encountering a torted testis, 221/340 (65%) will detort the testis, apply warm swabs and reassess after a time; 109/340 (32%) respondents additionally ask the anaesthetist to increase the oxygen flow. When asked how long they would wait to assess after detorsion, the majority of respondents (236/340; 69%) indicate a time between 5 and 15min, whereas 101/340 (30%) wait 15–20+min. (Supplementary Table S2 available online).

On detorting a viable testis, 300/340 (88%) use the same fixation method irrespective of age or pubertal stage, 22/340 (6%) respondents vary their method based on pubertal stage and 18/340 (5%) vary based on age. Those varying their approach with age typically report using sutureless methods such as dartos pouches for younger patients, and sutured fixation for pubertal or teenage boys.

**Figure 2:**
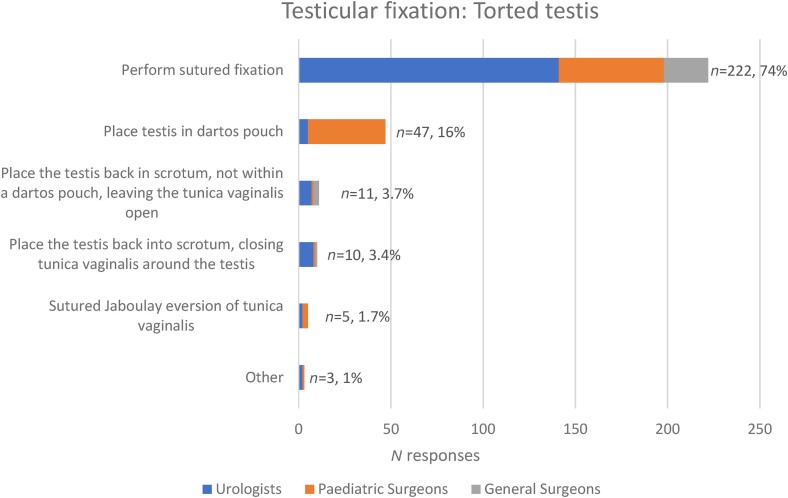
Management preference on finding a torted testis

**Figure 3:**
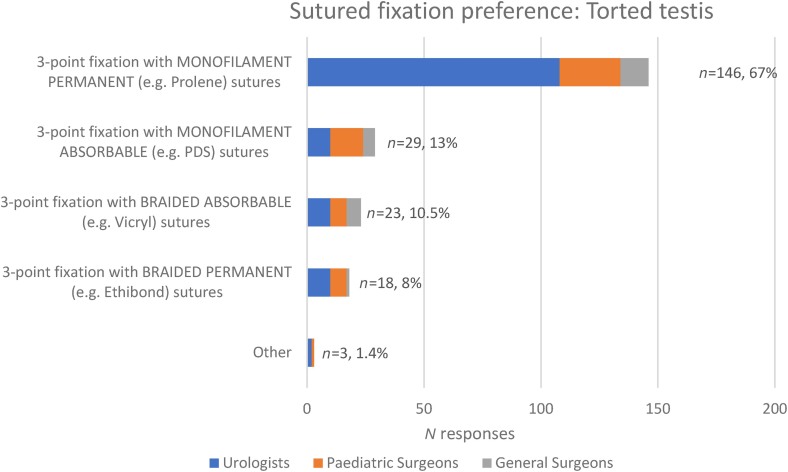
Preference of suture material for fixation of a torted testis

Of the 300/340 respondents opting for the same fixation method for detorted testes, 2 did not select a method. Of the other 298, sutured fixation of a detorted testis is undertaken by 222/298 (74%) of respondents (Figure 2), with 164/222 (74%) selecting nonabsorbable sutures (Figure 3). The next most common fixation method is a dartos pouch (47/298, 16%), which is almost exclusively used by paediatric surgeons.

If a nonviable testis is removed, 310/336 (92%) (4/340 surgeons were unable to answer this question because of a software error) respondents do not offer a prosthesis insertion in the same operation. Of the 26 surgeons who do, all only offer if the patient is pubertal or postpubertal. More urologists offer prosthesis in the same operation than paediatric surgeons or general surgeons (11% [95% CI 9–14]) vs 2% [95% CI 0–5] vs 7% [95% CI 4–10]).

When asked about contralateral exploration in cases of torsion, 332/340 (98%) perform this in all cases with identical fixation method to the ipsilateral side in most cases.

### Wound closure and postprocedure care

Most surgeons indicate that local anaesthetic is routinely infiltrated during the procedure (325/340, 96%). Closure of the incision in layers (295/340, 87%), with a continuous absorbable suture (232/295, 79%), followed by an interrupted absorbable skin suture (207/295, 70%) is the preferred choice of most respondents, the remainder opting for a single-layer closure (Supplementary Table S3 available online). The provision of a scrotal support varies between specialties, with urologists using them for all patients more frequently than paediatric surgeons. A significant proportion of specialists vary their approach based on age or pubertal stage, providing them for older or pubertal/postpubertal patients. Few surgeons change their approach to providing a scrotal support based on diagnosis (Supplementary Table S4 available online).

### Recurrent torsion

Of the 333/340 respondents that answered the question, the majority have not experienced a recurrent torsion in their career (222/333, 67%); 83 report encountering between one and five. Other responses indicated a possible misinterpretation of the question as simple ‘reoperation’ because 16 responses report higher numbers of 25–100, and when asked about the operation findings, responses such as ‘no torsion’ and ‘normal testis’ were common.

Responses regarding operation findings were analysed. Vague, nonspecific reports, and those not reporting recurrent torsion as mentioned above were excluded. Detailed reports of recurrent torsions were described in the context of multiple different previous fixation methods, such as nonabsorbable, absorbable sutures and sutureless (Supplementary Table S5 available online).

### Outcomes of importance for future research

**Figure 4:**
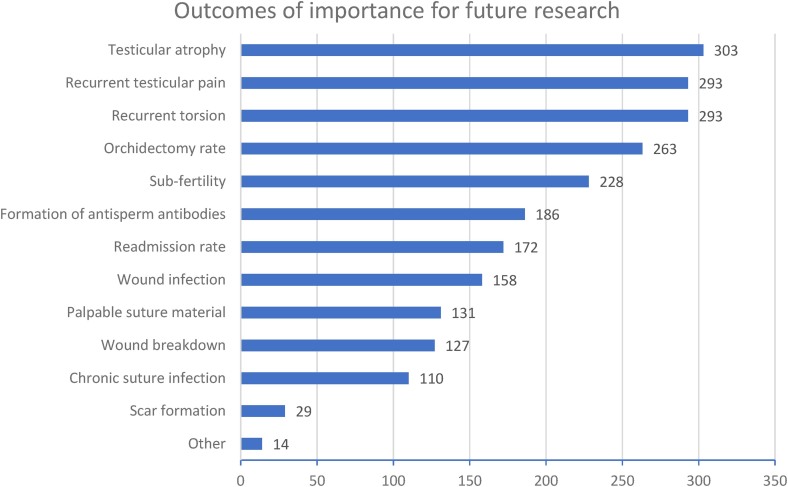
Respondents’ preference for most important outcomes for future research into emergency testicular fixation (checkbox – multiple preferences allowed)

The three most common outcomes that surgeons considered important for future research in emergency scrotal explorations were: testicular atrophy (303/340, 89%), recurrent torsion (293/340, 86%) and recurrent testicular pain (293/340, 86%) (Figure 4).

## Discussion

This study represents the largest survey of practice for emergency scrotal explorations, and the only one with scope across different surgical subspecialties. It has identified several key trends across different specialties. Based on the responses, a ‘typical’ methodology for emergency scrotal exploration in the UK is to use either a midline or transverse hemiscrotal incision. A nontorted testis is typically placed back into the scrotum, not sutured, and the contralateral side would not be explored. A torted testis would typically be untwisted, warm swabs applied for 5–15min, and if viable, a bilateral three-point fixation with monofilament nonabsorbable sutures would be performed. This would also typically be the case for a nontorted bell-clapper. A prosthesis is not typically offered at the same operation. Local anaesthetic is administered and the wound is closed in layers with absorbable sutures. Older patients are offered a scrotal support. These findings of current practice were consistent with the consensus statements derived from the BURST–BAUS FIX-IT study.^5^

The described approach reflects the adult practice, as the majority of the respondents were adult specialists. Key differences in the paediatric surgeons’ methodology were noted; for example, the almost exclusive use of transverse incisions, and a higher tendency to use sutureless fixation techniques, primarily the dartos pouch. The reasons for this variation in practice are beyond the scope of this survey. However, it may be because of differences in the anatomy of paediatric and adult testes/scrotums; for example, the latter may be less amenable to the use of dartos pouches because of the risk of bleeding while developing the subdartos plane. Subsequently, training for this procedure passed down within specialties is likely to be influenced by the anatomical considerations. The proportion of paediatric surgeons relying on sutureless fixation in torsion was higher than in a 2006 BAPS survey of 97 paediatric surgeons, in which only 15% favoured this technique.^8^ Neither this nor the current survey describe all paediatric surgeons, but may be representative of a trending change in practice over time.

Participants were selected based on their role and therefore likelihood of needing to perform emergency scrotal exploration within UK surgical practice. Trainee surgeons were included because they are often in the position of leading the surgery without a consultant present and will therefore likely have their own preference on fixation methods.

The majority of respondents performed emergency scrotal explorations on children (<16 years). Given that there are relatively few specialised paediatric surgery services across the UK, it is not unreasonable to assume that a large proportion of the paediatric caseload for this procedure is undertaken by those with a primarily adult interest. Several respondents indicated that they would change their approach based on the pubertal stage or age of the patient. For example, transverse hemiscrotal incision appears to be the approach of choice for younger patients, with midline favoured for older patients. This theme is continued, with some respondents using dartos pouches for fixation in children, and preferring suturing for older patients. This is again reflected in the difference in practice between adult and paediatric specialists.

The BURST–BAUS FIX-IT consensus statement suggested that a nontorted testis should not be fixed, and the contralateral side should not be explored, with the exception of the finding of a bell-clapper deformity, in which case bilateral fixation should be performed. This statement is in keeping with the practice noted in this survey, with the majority of respondents preferring this. The FIX-IT consensus does not state whether dartos pouch, the preference of a significant portion of respondents for use with a nontorted testis, was considered to be an unfavoured method. Dartos pouch was considered to be acceptable fixation for paediatric cases of torsion, but not adult patients. This was again reflected in the responses of the present survey.^5^

The aim of fixing a testis by any method is to prevent instances of future torsion. The results of this survey indicate a significant proportion of paediatric surgeons consider sutureless fixation methods such as dartos pouch adequate to prevent torsion even in confirmed cases. This is supported by data from animal studies in which fixation is achieved with this method by the formation of scar tissue; similarly, eversion of the TV is thought to produce a similar result. Other animal studies note damage to seminiferous tubules, decreased sperm production and atrophy induced by sutured fixation of the testis.^9–11^ This does not, however, appear to translate in human studies of testicular torsion into a reduction in paternity rates compared with the general population, regardless of whether the testis was removed or fixed.^12,13^ To date there have been no primary studies directly relating testicular fixation method to fertility.^14^ Uncertainty regarding this, in addition to known complications such as suture sinus and granulomas, could potentially be the reason that some surgeons prefer to avoid sutured fixation.

Testicular atrophy, recurrent testicular pain and recurrent torsion were highlighted as key areas for future research into testicular fixation. The former two could conceivably be influenced by unnecessary sutured fixation, when looked at in the context of animal data. A perceived benefit of sutured fixation is to prevent recurrent torsion. The data from this survey suggests that this is a very rare event, given the large volumes of procedures performed by each surgeon, and the fact the majority have never experienced this, despite a substantial proportion describing re-explorations for recurrent pain where nontorted testes were found. A common theme on the rare occasion when re-torsion was found is a lack of adhesions, and occurrence regardless of the previous method of fixation or suture material.

### Study limitations

The strengths of this survey are that the full spectrum of clinicians undertaking scrotal exploration are included with a national distribution. Limitations of the study are of selection bias and that the accuracy of the responses is based on participants’ correct interpretation of the question. The respondents may have a different practice from nonrespondents, so the sample may not reflect the true UK practice. Definite misinterpretations were noted in the question on findings in recurrent torsion. Questions about a particular practice are open to vulnerability, because a surgeon’s preference for a practice may change over time, and be influenced by new mentors, colleagues and working environments. It was also not possible to calculate an exact response rate because of the multiple modalities used to distribute the survey (direct to trusts and email distribution lists); however, electronic multimodality surveys, including the use of social media, are gaining acceptance in evidence-based medicine as effective ways to gather information.^15^

## Conclusions

In conclusion, this survey has illustrated a sample of current UK practice for emergency testicular fixation and some key difference across specialties. Most UK practitioners would not use sutured fixation for a nontorted testis, with the exception of a bell-clapper deformity. In cases of testicular torsion, sutured fixation is the most frequently chosen method; however, paediatric surgeons tend to use sutureless techniques for younger patients. Recurrent torsion is rare, and anecdotal evidence from this survey suggests a key factor may be a lack of adhesions. As a commonly performed emergency procedure but with complications of interest that are likely exceedingly rare, scrotal exploration and testicular fixation may benefit from a registry to monitor outcomes, and standardisation of techniques within and across specialties, to minimise variation in patient care and to streamline training in the procedure for junior surgeons.
